# Between-Session Reliability and Agreement of Body-Plethysmographic Lung Volumes in a Legacy Pulmonary Function Cohort

**DOI:** 10.3390/jcm15197704

**Published:** 2026-10-05

**Authors:** Carmine Izzo, Alessandro Izzo, Pasquale Mone, Simona Musto, Michele Ciccarelli, Francesco Giallauria

**Affiliations:** 1 Department of Medicine, Surgery and Dentistry, University of Salerno, 84081 Salerno, Italy; cizzo@unisa.it (C.I.); simusto@unisa.it (S.M.); mciccarelli@unisa.it (M.C.); 2 Department of Respiratory Diseases, San Giuseppe Moscati Hospital, 83100 Avellino, Italy; aleizzo85@yahoo.it; 3 Department of Medicine and Health Sciences ‘Vincenzo Tiberio’, University of Molise, 86100 Campobasso, Italy; pasquale.mone@unimol.it; 4 Casa di Cura “Clinica Montevergine”, 83013 Mercogliano, Italy; 5 Department of Translational Medical Sciences, Federico II University of Naples, Via S. Pansini 5, 80131 Naples, Italy

**Keywords:** body plethysmography, total lung capacity, residual volume, functional residual capacity, reliability, agreement, between-session variability, retrospective study

## Abstract

**Background:** Serial interpretation of lung volumes requires assessing both reliability and agreement. We examined these properties in a historical clinical dataset. **Methods:** This retrospective secondary analysis included 72 participants, excluding seven same-session pre/post-bronchodilator pairs. The primary analysis used 19 separately dated pairs labelled pre-bronchodilator at both examinations; the expanded 65-pair analysis was exploratory. Documented intervals were two to three days; the investigator retrospectively confirmed separate-day examinations within six days for all 65 pairs. Total lung capacity (TLC) and residual volume (RV) were principal outcomes; functional residual capacity was secondary and available in the same 19 participants. Analyses included two-way mixed-effects, absolute-agreement, single-measure intraclass correlation coefficients (ICCs), Lin’s concordance correlation coefficient, Gaussian reference limits, empirical difference quantiles, and leave-one-out analyses. **Results:** Fourteen primary participants had recorded chronic obstructive pulmonary disease labels without independently verified diagnostic post-bronchodilator spirometry. Primary TLC and RV ICCs were 0.947 (95% bootstrap confidence interval 0.838–0.988) and 0.940 (0.818–0.974), respectively. Mean second-minus-first differences were −0.033 and −0.015 L, with Gaussian reference limits of −0.964 to 0.898 L and −0.934 to 0.904 L, respectively. Corresponding empirical 2.5th–97.5th percentiles were −1.122 to 0.641 L and −0.875 to 0.803 L. Removing one observation reduced the standard deviation of paired differences by up to 28.8% for TLC and 14.8% for RV. **Conclusions:** High reliability estimates coexisted with appreciable paired variation. Unverified technical quality and clinical stability preclude attributing this variation solely to measurement error or establishing clinical response thresholds.

## 1. Introduction

Measurement of static lung volumes complements spirometry by quantifying total lung capacity (TLC), functional residual capacity (FRC), and residual volume (RV) [1,2]. Interpretation relative to appropriate reference values helps characterize restrictive ventilatory impairment, gas trapping, and hyperinflation, but requires clinical context and does not by itself establish a disease diagnosis [3,4]. The 2023 European Respiratory Society/American Thoracic Society (ERS/ATS) technical statement emphasizes linked spirometry, manoeuvre acceptability and within-session repeatability, equipment calibration, and biological quality control [2].

Serial assessment requires distinguishing reliability from agreement. A high Pearson correlation does not establish that repeated measurements are sufficiently close for individual clinical interpretation [5]. Reliability, summarized by an appropriately specified intraclass correlation coefficient (ICC), reflects between-participant variability relative to total measurement variability, whereas Lin’s concordance correlation coefficient (CCC) assesses concordance with the identity line [6,7]. Agreement can also be examined in the original measurement units using the mean paired difference and estimated limits of agreement (LoA) [5,8]. High reliability or concordance coefficients can therefore coexist with appreciable differences between repeated measurements. Interpretation also depends on the measurement conditions and the interval between assessments [8].

Earlier work reported lower coefficients of variation for TLC than for FRC across consecutive plethysmographic measurements and identified differences between observers reading the same tracings [9]. An ERS/ATS workshop report subsequently emphasized that reproducibility estimates depend on the measurement technique and population, and recommended laboratory-specific assessment of between-day reproducibility [10]. In patients with airflow obstruction and hyperinflation, reductions in RV and FRC have been documented after bronchodilation [11]. Such treatment-related changes should be distinguished from the variation observed between repeated assessments under comparable conditions.

We therefore conducted an exploratory retrospective secondary analysis of a recovered legacy pulmonary-function dataset. The main objective was to characterize between-session reliability and agreement for TLC and RV measured in liters. The 19 participants with dataset-documented separate visits were designated as the primary analysis population; the expanded population of 65 participants, including those 19, was retained for exploratory analyses. Secondary outcomes included spirometric measures and FRC, with paired FRC available only in the primary population.

## 2. Materials and Methods

### 2.1. Study Design and Analysis Populations

**Figure 1:**
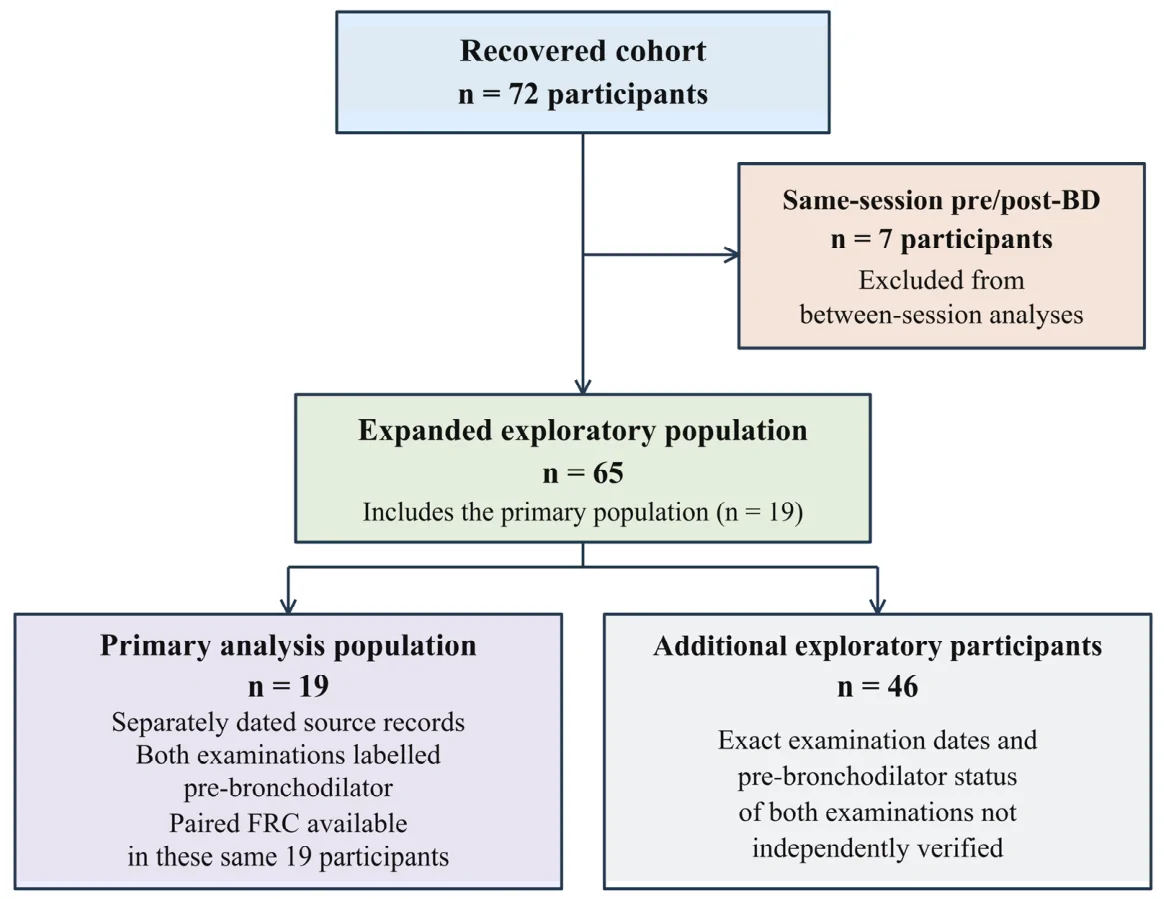
Participant flow and analysis populations. Paired TLC, RV, FEV_1_, FVC and FEV1/FVC values available for all 65 participants. Paired FRC values available only for the 19 primary participants. BD, bronchodilator; TLC, total lung capacity; RV, residual volume; FRC, functional residual capacity; FEV_1_, forced expiratory volume in one second; FVC, forced vital capacity.

This was an exploratory retrospective secondary analysis of a fixed legacy pulmonary-function dataset. The dataset had been compiled earlier and contained no direct or indirect patient identifiers (e.g., no name, date of birth, medical record number, or address). The present investigators received and analysed only this pre-existing, non-identifiable dataset and had no access to the original identifiable clinical records from which it had been derived, nor to any key permitting re-identification of participants. According to the dataset thesis, pulmonary-function examinations were performed between December 2014 and May 2016. The recovered cohort comprised 72 participants with recorded labels of COPD (*n* = 60), interstitial lung disease (*n* = 7), or healthy status (*n* = 5); these labels were not independently adjudicated. All examinations, including repeat assessments, were performed for clinical care and were not prescribed for the present analysis. Seven participants had explicitly labelled same-session pre/post-bronchodilator measurements and were excluded from all between-session analyses, although their records were retained in the dataset. The remaining 65 participants formed the expanded exploratory population (Figure 1).

The 19 participants with separately dated entries in the dataset labelled pre-bronchodilator at both examinations were designated as the primary analysis population. Eighteen pairs were two days apart and one pair was three days apart. This documentation-based definition did not imply verified technical acceptability of the examinations. All 65 retained pairs represented examinations performed on different days, no more than six days apart. For the 46 additional participants, comparable bronchodilator conditions were reported, but exact examination dates and pre-bronchodilator status at both examinations could not be independently verified from the dataset’s available records. Missing dates and exact intervals were not imputed. Paired numerical FRC values were recoverable only from the same separately dated dataset defining the primary population; therefore, the FRC subset and the primary population comprised exactly the same 19 participants. FRC was treated as a secondary outcome.

Sample size was determined by the available historical records; no prospective sample-size calculation was performed. Reporting was guided by the Guidelines for Reporting Reliability and Agreement Studies (GRRAS) [8]. GRRAS reporting items were only partially fulfilled because operator information, technical-quality documentation, blinding, and independence of measurements were not recorded in the dataset and could not be reconstructed. The item-by-item reporting audit in Supplementary Table S4 distinguishes information recorded in the dataset, retrospective investigator clarification, and unavailable information.

### 2.2. Pulmonary Function Measurements

According to the dataset thesis, spirometry and body plethysmography were performed using the same MasterScope Body system (JAEGER, Hoechberg, Germany), with a “Spirometry Filter 74” mouthpiece. Separate-day testing within six days for all 65 retained pairs and reported comparable bronchodilator conditions for the 46 additional participants, as distinguished from the dataset-documented pre-bronchodilator labels in the primary population (Section 2.1). The investigator reported no intervening clinical changes, while unchanged usual treatment was based on patients’ accounts. Drug-specific regimens, actual administration or withholding, exacerbations, hospitalization, antibiotic exposure, surgery, pre-test smoking, physical activity, and time of day could not be independently verified from the available records.

The primary outcomes were TLC and RV in liters. Secondary outcomes were FEV_1_, FVC, and FRC in liters and the recorded FEV_1_/FVC ratio expressed as a percentage on a 0–100 scale; paired changes in this ratio were expressed in percentage points. Recorded FEV_1_/FVC ratios were retained rather than recalculated from rounded volume values. Paired numerical FRC values were available only for the same 19 participants constituting the primary population. Missing FRC values were not reconstructed or imputed.

Recovered dataset carried the historical reference labels “ERS 1993 Update” and “Zapletal”, but the implemented equations and software version could not be verified. Legacy percent-predicted values were not included in the analyses, which focused on absolute volumes and recorded ratios. GLI static-lung-volume z-scores were not recalculated. Although age, sex, and height were available for the 19 primary participants, applicability of the GLI reference population was not documented, and the observed age range extended beyond the published 5–80-year reference range [3]. The corresponding demographic information was unavailable for the 46 additional participants.

COPD and interstitial lung disease were treated as recorded diagnostic labels rather than independently adjudicated diagnoses. Diagnostic post-bronchodilator confirmation of COPD could not be independently verified; pre-bronchodilator report labels were not considered evidence of such confirmation. No GOLD grades or reference-based categories of hyperinflation were assigned.

Software version, calibration logs, manoeuvre counts, acceptability assessments, quality grades, within-session repeatability, and session-level operator identity, number, and training records were unavailable. Blinding and independence of measurements could not be verified. Consequently, adherence to contemporaneous or current ERS/ATS technical standards could not be retrospectively established [1,2]. The observed between-session differences could not be partitioned into technical, biological, and potentially clinical components.

### 2.3. Outcomes and Estimands

For each outcome, the paired difference was defined as D2 − D1. Reliability was summarized using a two-way mixed-effects, absolute-agreement, single-measure ICC [ICC(A,1)], with participants treated as random and the two observed occasion positions treated as fixed [7,12]. Absolute agreement was selected so that systematic differences between occasions counted against agreement; the target was a single session-level measurement, not the average of two sessions. The ICC describes differentiation between participants relative to observed variability. Interpretation was restricted to the observed occasion structure, without estimating reliability across operators or laboratories.

Lin’s CCC summarized concordance with the identity line by combining linear association with differences in paired means and variances [6,13]. Unlike Pearson correlation, it penalizes departures from identity. It complements the ICC’s variance-model formulation, but neither coefficient directly expresses the range of individual differences in physical units.

Bland–Altman analysis characterized paired differences relative to pair means [5]. Bias was the arithmetic mean difference, and Gaussian reference limits were calculated as bias ± 1.96 × SD_diff, where SD_diff is the sample standard deviation of the paired differences. These limits approximate a central 95% range of individual differences only under suitable distributional and sampling assumptions; they are not confidence intervals for the mean difference. Empirical 2.5th and 97.5th percentiles were also reported. Neither approach was considered to establish 95% coverage for future observations. Clinical interchangeability was not inferred because no independently justified acceptable-difference criterion was available. Proportional bias was explored by regressing paired differences on pair means.

Descriptive dispersion was additionally summarized as S_w = SD_diff/√2 and D_95_ = 1.96 × SD_diff. Thus, D_95_ is the half-width of the Gaussian reference limits around the mean bias, not the 95th percentile of absolute differences. The descriptive coefficient of variation was calculated as 100 × S_w divided by the grand mean of the D1 and D2 measurements.

Under stable conditions and an appropriate independent Gaussian measurement-error model, MDC_95_ addresses whether an individual change exceeds expected measurement error at the nominal 95% level; it does not establish clinical importance or assign a 95% probability that a particular response is physiological [14,15]. Because clinical stability and technical control could not be independently verified here, S_w and D_95_ were used only as descriptive measures of observed variation, with inseparable technical, biological, and potentially clinical components [16]. D_95_ was not interpreted as a validated MDC_95_ or clinical response threshold. These quantities concern individual paired differences, not the precision of a group-average change.

### 2.4. Statistical Analysis

Continuous variables were summarized using means and standard deviations, with medians and ranges where appropriate; categorical variables were summarized using counts and percentages with the relevant denominators. Analyses used complete D1–D2 pairs for each outcome. Missing values were not imputed.

The Shapiro–Wilk test was applied to within-participant differences for each outcome and analysis population. When the Shapiro–Wilk *p* value was ≥0.05, a paired *t*-test was used, with a model-based 95% confidence interval for the mean difference. Otherwise, a Wilcoxon signed-rank test was used, and the Hodges–Lehmann pseudomedian was calculated as the median of the Walsh averages of the paired differences. All signed-rank analyses used normal approximations with continuity correction for *p* values and confidence intervals. Non-rejection of the Shapiro–Wilk test was not interpreted as proof of normality, and signed-rank inference remained subject to its symmetry assumption. Shapiro–Wilk results and the corresponding test selections are provided for all outcomes in Supplementary Materials S1.

Uncertainty in ICC, CCC, mean bias, dispersion statistics, and the endpoints of both Gaussian reference limits and empirical limits was summarized using 95% percentile bootstrap confidence intervals based on 5000 participant-level resamples. Resampling was performed within each analysis population and outcome, preserving each participant’s D1–D2 pair. Dispersion statistics included SD_diff, S_w, D_95_, and the descriptive coefficient of variation. Empirical limits and bootstrap confidence limits were calculated using R’s type-7 quantiles. Bootstrap confidence intervals for mean bias were distinct from the confidence intervals accompanying the paired location tests. The choice of a rank-based location test did not validate Gaussian limits for individual differences; neither empirical quantiles nor bootstrap uncertainty established 95% coverage for future observations.

The primary analysis included 19 participants. Exploratory analyses applied the same methods to the expanded population of 65 participants and to participants with a recorded COPD label within the primary and expanded populations (n = 14 and n = 56, respectively). These populations overlapped, and comparisons of their estimates were descriptive. No independent-group tests or severity-adjusted analyses were performed. The expanded FRC analysis contained the same 19 participants as the primary analysis and was not treated as independent replication. Leave-one-out analyses assessed the influence of individual pairs on D_95_ and on Gaussian and empirical limits by omitting each participant in turn. All eligible pairs were retained in the principal estimates.

All analyses were exploratory. Paired location tests and tests of proportional-bias slopes were two-sided, and no multiplicity adjustment was applied. Interpretation emphasized effect estimates and their uncertainty; nonsignificant location tests were not considered evidence of equivalence or acceptable individual agreement. Manuscript *p* values are reported to three decimal places or as *p* < 0.001, with full numerical precision retained in the supplementary statistical outputs.

Analyses were performed in R version 4.6.1, with the bootstrap random seed set to 20260912. Supplementary Materials S1 contains aggregate results, variable definitions, analysis code, and instructions.

### 2.5. Ethical Considerations

This study was a retrospective secondary analysis of a pre-existing, non-identifiable pulmonary-function dataset. The dataset contained no direct or indirect patient identifiers, and the investigators had no access to, and did not seek, the original identifiable clinical records or any key permitting re-identification of participants. Accordingly, the dataset used for the present analysis did not constitute “personal data” as defined in Article 4(1) of Regulation (EU) 2016/679 (GDPR) and Recital 26 thereof, and the processing performed for this secondary analysis fell outside the scope of the GDPR and of the research-ethics-review requirements, including paragraph 32 of the Declaration of Helsinki, which specifically addresses the use of identifiable human data or material, that apply to research involving identifiable human subjects. On this basis, ethical review and approval by an Institutional Review Board/Ethics Committee were not sought for this analysis. The general principles of the Declaration of Helsinki concerning respect for participants and confidentiality nonetheless guided the conduct of this study. All original pulmonary-function examinations, including repeat assessments, were performed exclusively for clinical care and were not prescribed for research purposes; no additional examinations, interventions, or changes in clinical management were undertaken for this analysis. Patients had provided written consent, at the time of clinical admission and before any procedures, for the future scientific use of their clinical data and the dissemination of aggregate research findings, under the standard practice of the originating institution; this consent preceded and was not specific to the present secondary analysis. A.O.R.N. dei Colli–Monaldi Hospital, Naples, the institution from which the original data derive, was informed of this use of the data.

## 3. Results

### 3.1. Cohort and Data Availability

The recovered cohort comprised 72 participants. Seven participants had explicitly labelled same-session pre/post-bronchodilator measurements and were excluded from the between-session analyses, leaving 65 retained participants. The primary population consisted of 19 participants with dataset-documented separate-day examinations: 14 (73.7%) had a recorded COPD label, two (10.5%) a recorded interstitial lung disease label, and three (15.8%) were recorded as healthy; 11 (57.9%) were male. The expanded exploratory population included these 19 participants and 46 additional participants. Among the 65, 56 (86.2%) had a recorded COPD label, five (7.7%) a recorded interstitial lung disease label, and four (6.2%) were recorded as healthy; 48 (73.8%) were male (Table 1).

Age, height, weight, and calculated BMI were available for 26 of the 72 recovered participants and for all 19 primary participants. Within the expanded population, these demographic data were available only for the same 19 primary participants. Mean age was 60.7 years (SD 15.4; range 28–83) among the 26 participants with available data and 61.7 years (SD 16.1; range 28–83) in the primary population. Available-case anthropometric summaries and their denominators are reported in Table 1. Detailed information on smoking, medication regimens, comorbidities, and independently verified disease severity was unavailable. Baseline spirometry and lung-volume measurements, including RV/TLC, are reported in Supplementary Table S3.

The median between-session interval recorded in the dataset was two days (range two to three) in the 19 primary participants. According to the principal investigator’s retrospective account, all 65 retained pairs represented examinations on different days within six days; exact dates remained unavailable for the additional 46 participants. Paired TLC, RV, FEV_1_, FVC, and FEV_1_/FVC values were complete in all 65 retained participants. Numerical FRC values at both visits were available only for the same 19 participants constituting the primary population; no additional paired FRC observations were available in the expanded population.

### 3.2. Primary Outcomes: TLC and RV

In the primary population of 19 participants, mean TLC was 6.04 L (SD 1.44) at D1 and 6.01 L (SD 1.41) at D2. The Shapiro–Wilk *p* value for paired differences was 0.022. The Hodges–Lehmann pseudo median difference was 0.010 L (95% asymptotic CI −0.180 to 0.170; *p* = 0.763). ICC(A,1) was 0.947 (95% bootstrap CI 0.838–0.988), and Lin’s CCC was 0.944 (95% bootstrap CI 0.831–0.988) (Table 2).

Mean RV was 3.02 L (SD 1.30) at D1 and 3.01 L (SD 1.35) at D2. The Shapiro–Wilk *p* value was 0.311, and the paired *t*-test yielded a mean difference of −0.015 L (95% CI −0.241 to 0.211; *p* = 0.893). ICC(A,1) was 0.940 (95% bootstrap CI 0.818–0.974), and Lin’s CCC was 0.937 (95% bootstrap CI 0.809–0.973).

**Figure 2:**
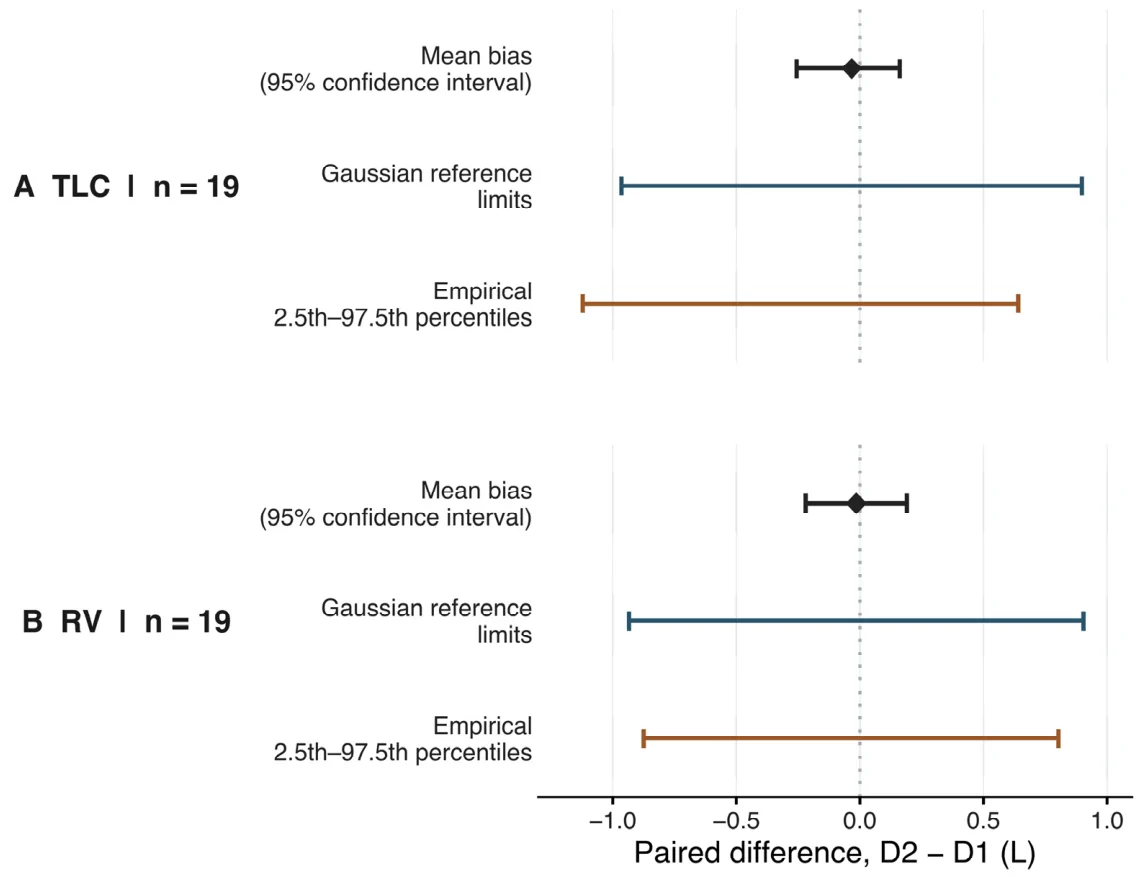
Aggregate agreement summaries for TLC (**A**) and RV (**B**) in the primary population (n = 19). Differences are D2 − D1, in liters. The vertical dotted line at 0 L indicates no difference between the second and first examinations (D2 − D1 = 0). Black diamonds show mean bias, with black bars indicating its 95% percentile-bootstrap CI. Blue bars span the Gaussian reference limits (mean bias ± 1.96 × SD_diff_), and orange bars span the empirical 2.5th–97.5th percentiles (type-7 quantiles). Blue and orange bars describe the spread of paired differences, not confidence intervals for a mean. Endpoint CIs are reported in Table 3. Participant-level observations are not displayed. These ranges do not establish 95% coverage for future differences or clinical interchangeability. CI, confidence interval; RV, residual volume; TLC, total lung capacity.

The arithmetic mean biases were −0.033 L for TLC and −0.015 L for RV. Gaussian reference limits were −0.964 to 0.898 L for TLC and −0.934 to 0.904 L for RV. The corresponding empirical 2.5th and 97.5th percentiles were −1.122 and 0.641 L for TLC and −0.875 and 0.803 L for RV. Bootstrap confidence intervals for mean bias and for each limit are reported in Table 3; aggregate agreement summaries are shown in Figure 2.

**Figure 3:**
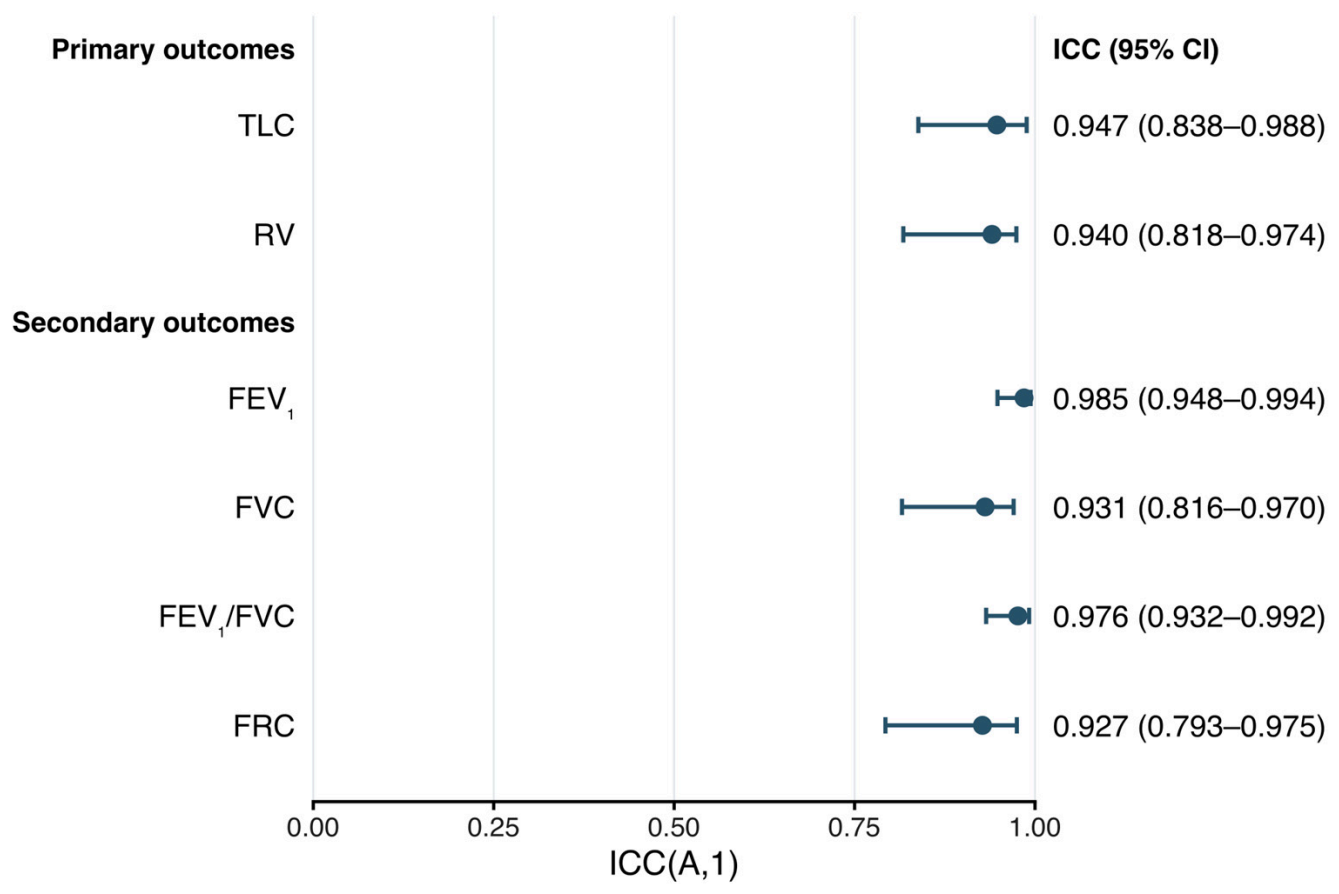
Absolute-agreement, single-measure intraclass correlation coefficients [ICC(A,1)] in the primary population. Points show the estimates and horizontal bars their 95% percentile-bootstrap CIs from 5000 participant-level resample. All six outcomes are based on the same 19 participants. The two-way mixed-effects model treats participants as random and the two observed occasion positions as fixed. TLC and RV are primary outcomes; the other outcomes are secondary. ICC is dimensionless and does not quantify the magnitude of individual differences in physical units. CI, confidence interval; FEV_1_, forced expiratory volume in one second; FRC, functional residual capacity; FVC, forced vital capacity; RV, residual volume; TLC, total lung capacity.

Descriptive D_95_ was 0.931 L (95% bootstrap CI 0.420–1.323) for TLC and 0.919 L (95% bootstrap CI 0.565–1.183) for RV. The corresponding descriptive S_w values were 0.336 and 0.332 L, and descriptive coefficients of variation were 5.6% and 11.0%, respectively. Exploratory regression tests for proportional bias yielded *p* values of 0.797 for TLC and 0.678 for RV. ICC estimates and their uncertainty for all six outcomes in the primary population are summarized in Figure 3.

### 3.3. Secondary Spirometric Outcomes

In the primary population of 19 participants, ICC(A,1) was 0.985 for FEV_1_ (95% bootstrap CI 0.948–0.994), 0.931 for FVC (0.816–0.970), and 0.976 for FEV_1_/FVC (0.932–0.992). Paired comparisons and concordance estimates are reported in Table 4; ICC estimates and their uncertainty are also shown in Figure 3.

The mean FEV_1_ difference was 0.036 L (95% CI −0.037 to 0.109; *p* = 0.318). The Hodges–Lehmann FVC pseudo median difference was −0.048 L (95% asymptotic CI −0.200 to 0.120; *p* = 0.513), whereas the arithmetic mean FVC bias was −0.057 L. The mean FEV_1_/FVC difference was 1.579 percentage points (95% CI −0.430 to 3.588; *p* = 0.116).

Descriptive D_95_ was 0.298 L for FEV_1_ (95% bootstrap CI 0.188–0.378), 0.570 L for FVC (0.414–0.660), and 8.168 percentage points for FEV_1_/FVC (5.027–10.657). Mean biases, Gaussian reference limits, empirical percentiles, and associated uncertainty intervals are reported in Table 5. Exploratory regression tests for proportional bias yielded *p* values of 0.319 for FEV_1_, 0.605 for FVC, and 0.996 for FEV_1_/FVC.

### 3.4. FRC Analysis

Paired numerical FRC values were recoverable only from the same separately dated dataset defining the primary population (n = 19). No additional FRC pairs were available in the expanded population. Consequently, FRC estimates across these populations described the identical 19 pairs and did not constitute independent replication.

Mean FRC was 4.13 L (SD 1.39) at D1 and 4.11 L (SD 1.53) at D2. The Shapiro–Wilk *p* value for paired differences was 0.068, and the paired *t*-test yielded a mean difference of −0.018 L (95% CI −0.294 to 0.257; *p* = 0.890). ICC(A,1) was 0.927 (95% bootstrap CI 0.793–0.975), and Lin’s CCC was 0.923 (95% bootstrap CI 0.784–0.973) (Table 4).

Gaussian reference limits were −1.140 to 1.103 L, and the empirical 2.5th and 97.5th percentiles were −1.300 and 0.840 L, respectively. Descriptive S_w was 0.405 L, descriptive D_95_ was 1.122 L (95% bootstrap CI 0.559–1.568), and the descriptive coefficient of variation was 9.8%. Bootstrap confidence intervals for mean bias, limit endpoints, and dispersion statistics are reported in Table 5. The exploratory regression test for proportional bias yielded *p* = 0.320.

### 3.5. Exploratory and Sensitivity Analysis

Comparisons with the expanded exploratory population (n = 65, including all 19 primary participants) were descriptive because the populations overlapped. For TLC, ICC(A,1) was 0.947 in the primary population and 0.917 in the expanded population, with corresponding descriptive D_95_ values of 0.931 and 1.081 L. For RV, ICC(A,1) was 0.940 and 0.883, respectively, and descriptive D_95_ was 0.919 and 1.091 L. The direction differed for spirometry outcomes: primary versus expanded D_95_ was 0.298 versus 0.225 L for FEV_1_, 0.570 versus 0.463 L for FVC, and 8.168 versus 7.267 percentage points for FEV_1_/FVC. FVC ICC(A,1) was lower in the primary population (0.931 versus 0.948). Expanded-population estimates and their uncertainty are reported in Supplementary Table S1.

Among the 14 primary participants with a recorded COPD label, TLC ICC(A,1) was 0.940 (95% bootstrap CI 0.788–0.990) and RV ICC(A,1) was 0.927 (0.652–0.972); corresponding descriptive D_95_ values were 0.955 and 0.942 L. Among the 56 participants with a recorded COPD label in the expanded population, TLC and RV ICC(A,1) estimates were 0.910 (0.828–0.964) and 0.853 (0.717–0.936), with descriptive D_95_ values of 1.131 and 1.146 L, respectively (Supplementary Table S2). These exploratory subgroups were defined by recorded labels; diagnostic post-bronchodilator confirmation of COPD was unavailable.

In leave-one-out analyses of the primary population, descriptive D_95_ ranged from 0.663 to 0.958 L for TLC and from 0.783 to 0.946 L for RV. The largest reductions relative to the corresponding complete-sample estimates were 28.8% and 14.8%, respectively. For FRC, D_95_ ranged from 0.830 to 1.154 L, with a maximum reduction of 26.0%. In the expanded population, the maximum reductions for TLC and RV were 18.5% and 17.7%, respectively. Each sensitivity fit omitted one participant in turn; all eligible pairs remained in the principal estimates. Aggregate influence summaries are provided in Supplementary Materials S1.

## 4. Discussion

In this exploratory analysis of 19 dataset-documented between-session pairs, TLC and RV had ICC(A,1) point estimates of 0.947 and 0.940, respectively, with descriptive D_95_ values of 0.931 and 0.919 L. Central location estimates were close to zero, but this did not establish equivalence between sessions. FRC, a secondary outcome assessed in the same participants, had a descriptive D_95_ of 1.122 L. Dispersion estimates were sensitive to individual observations, and the observed variation could not be partitioned into technical, biological, and potentially clinical components. These findings characterize between-session agreement in this historical dataset; they do not establish isolated measurement error or validated thresholds for interpreting individual clinical change.

ICC(A,1) reflects differentiation between participants relative to variability in the measurements. Its absolute-agreement formulation penalizes systematic differences between sessions, but the coefficient remains influenced by the spread of participant values [7,8]. Lin’s CCC assesses closeness to the identity line by combining linear association with differences in means and variances, complementing the ICC’s variance-model formulation [6,13]. Neither coefficient directly expresses the magnitude or range of paired differences in litres or percentage points. Bland–Altman analysis complements these summaries by describing mean bias and the distribution of individual differences; a small mean bias does not imply that those differences are narrowly distributed [5]. In this dataset, Gaussian reference limits and empirical quantiles were descriptive summaries, not confidence intervals for a mean or validated 95% prediction intervals for future observations. Their bootstrap confidence intervals quantify sampling uncertainty without establishing future coverage. Clinical interchangeability could not be inferred because no independently justified acceptable-difference criterion was available [5].

Teculescu and colleagues reported lower variability for TLC than for FRC across five consecutive plethysmography measurements and differences between observers interpreting the tracings [9]. Hankinson and colleagues emphasized that within-subject variability should be characterized for each laboratory, measurement technique, and participant group [10]. In our primary population of 19 participants, the descriptive between-session CV was 5.6% for TLC, 11.0% for RV, and 9.8% for FRC. Differences in protocols, populations, and variability metrics preclude direct quantitative benchmarking against earlier studies. These CVs must also be distinguished from within-session quality criteria. The 2023 ERS/ATS body-plethysmography grading system specifies FRC repeatability within 5% for Grades A/B and within 10% for Grades C/D/U, calculated as the range of measurements divided by their mean [2]. For both FRC and linked slow vital capacity measurements, the manoeuvre-count criteria require at least three acceptable measurements for Grade A, at least two for Grades B/C, one acceptable plus at least one usable for Grade D, and no acceptable but at least one usable for Grade U; the overall test grade follows the lowest component grade [2]. These percentages describe the spread of measurements within a session, not between-session CVs or thresholds for individual clinical change. The required manoeuvre-level information was unavailable in our dataset, preventing retrospective assignment of quality grades or verification of compliance with these criteria.

The interpretation of an individual RV change must be distinguished from inference about a group-average bronchodilator response. For n independent participants, the standard error of the mean paired change is the standard deviation of the paired differences divided by √n. Consequently, appreciable dispersion of individual changes does not preclude a precisely estimated group-average response, and our descriptive dispersion estimates cannot be used to invalidate published group-level findings. Under stable conditions, with systematic bias accounted for and an appropriate independent Gaussian measurement-error model, MDC_95_ assesses whether an individual change exceeds expected measurement error at the nominal 95% level [14,16]. It does not assign a 95% probability that a particular response is physiological or establish that the change is clinically important; clinical importance requires separate clinical or patient-centred justification and depends on context [14,15]. In this study, short inter-examination intervals and patient-reported unchanged treatment did not independently establish the physiological stability and technical control required to isolate measurement error. D_95_ therefore describes observed between-session variation and is not a validated criterion for identifying individual treatment responses or clinically important change. Interpretation of serial RV measurements requires contemporaneous clinical context and technical-quality information.

The secondary FRC analysis involved exactly the same 19 participants as the primary TLC and RV analyses. No additional paired FRC observations were available in the expanded population; consequently, reporting FRC under both population definitions does not constitute independent replication. FRC ICC(A,1) was 0.927 (95% bootstrap CI 0.793–0.975), while Gaussian reference limits ranged from −1.140 to 1.103 L and descriptive D_95_ was 1.122 L (95% bootstrap CI 0.559–1.568). These estimates indicate appreciable uncertainty and sensitivity to individual observations: omitting the most influential pair reduced D_95_ by 26.0%, to 0.830 L. This influence analysis did not justify excluding that pair, and all observations were retained in the principal estimates. Contemporary standards emphasize acceptable FRC manoeuvres, linked spirometry, and equipment quality control [2]. The corresponding technical records were unavailable, preventing attribution of the observed dispersion to specific manoeuvre, operator, calibration, or software effects, or its separation from biological and potentially clinical variation.

Comparisons between the primary and expanded populations were exploratory and outcome-dependent. The 19 primary participants were included within the expanded population of 65, so these analyses were not independent replications. Primary TLC and RV had smaller D_95_ estimates than the expanded population (0.931 versus 1.081 L and 0.919 versus 1.091 L, respectively), together with higher ICC point estimates. In contrast, primary FEV_1_, FVC, and FEV_1_/FVC had larger D_95_ estimates, and FVC had a lower ICC. This mixed pattern does not establish that limited dataset documentation inflated variability, nor does it justify treating the expanded estimates as an upper bound. Differences in participant composition, selection, clinical circumstances, and documentation could not be disentangled. Appropriate reference equations remain important for physiological interpretation [2,3,4], but legacy percent-predicted results were excluded because their historical reference-equation implementation could not be verified. Age, sex, and height were available for all 19 primary participants, whereas age and height were unavailable for the additional 46. GLI static-lung-volume z-scores were not recalculated: applicability of the reference population was undocumented, and the primary age range extended beyond the published 5–80-year range [3]. The analyses therefore remained focused on absolute volumes and recorded ratios.

A broader methodological consideration is whether a repeated-measurement strategy is adequate for the decision it is intended to support. Mari and colleagues used a decision-analytic simulation to examine how serial blood eosinophil measurements influence detection of the eosinophil criterion for biologic-treatment eligibility in COPD [17]. In an editorial concerning a pirfenidone trial in progressive unclassifiable interstitial lung disease, Sgalla and colleagues discussed how home-spirometry variability compromised analysis of the primary endpoint [18]. These publications provide methodological analogies, not direct validation of body plethysmography or transferable thresholds, sampling schedules, or treatment effects. For the present findings, translation into individual-response criteria would require prospective evaluation with documented clinical stability, standardized technical quality, and independently justified clinical anchors.

## 5. Strengths and Limitations

Strengths include the explicit distinction between 19 dataset-documented primary pairs and an expanded exploratory population of 65, together with exclusion of seven identified same-session pre/post-bronchodilator pairs from between-session analyses. Paired TLC, RV, and spirometry values were complete in all 65 retained participants, allowing analyses without imputation. Complementary reliability and agreement statistics, empirical difference quantiles, participant-level bootstrap intervals, and leave-one-out analyses characterize uncertainty and observation sensitivity while retaining all eligible pairs in the principal estimates. Numerical completeness, however, does not establish technical validity.

The analysis relied on available historical clinical records rather than a prospectively designed reliability study. No dated prespecified analysis plan or prospective sample-size calculation was available. The small primary sample limits precision, and selection according to dataset-document availability may affect representativeness. All 65 retained pairs were separate-day examinations within six days, but exact dates and pre-bronchodilator labels at both examinations were dataset-documented only for the primary 19. The additional 46 had comparable bronchodilator conditions and no there was no intervening clinical changes, and of treatment changes. Software version, calibration logs, manoeuvre counts, acceptability, within-session repeatability, quality grades, and operator number, and identity were unavailable. Blinding and independence of the measurement process could not be established. Adherence to contemporaneous or current technical standards therefore could not be certified [1,2], and technical, biological, and potentially clinical contributions to variation remain inseparable. The GRRAS reporting audit identifies these gaps but represents only partial fulfilment of reporting requirements and cannot remedy the underlying design limitations [8] (Supplementary Table S4).

Diagnostic labels were not independently adjudicated. Fourteen primary participants had a recorded COPD label without independently verifiable diagnostic post-bronchodilator confirmation. The small groups labelled as interstitial lung disease or healthy did not support credible disease-specific comparisons. Detailed smoking, comorbidity, and medication-regimen data were unavailable, and disease severity could not be independently established. Consequently, the label-restricted analyses remain exploratory and do not establish COPD-specific, GOLD-specific, or severity-adjusted performance estimates.

Age, height, weight, and calculated BMI were available for all 19 primary participants but not for the additional 46; sex was available throughout. Historical reference-equation implementation could not be verified, so legacy percent-predicted analyses were omitted. GLI static-lung-volume z-scores were not recalculated because reference-population applicability was undocumented and the primary age range extended beyond the published 5–80-year range [3], not because demographic inputs were universally absent. FRC was a secondary outcome available in exactly the same 19 primary participants, with no additional paired observations in the expanded population.

Small-sample tail estimation and sensitivity to individual pairs limit interpretation of the dispersion estimates. Bootstrap confidence intervals and empirical quantiles do not establish 95% coverage for future observations. Multiple outcomes and subgroup analyses were examined without multiplicity adjustment, so nominal findings remain hypothesis-generating. No independent validation cohort or clinically justified change criterion was available; D_95_ therefore remains descriptive rather than a validated measurement-error or individual-response threshold.

## 6. Conclusions

In this exploratory analysis of 19 dataset-documented between-session pairs, TLC and RV showed high ICC point estimates alongside appreciable variation in individual paired differences. These findings emphasize the distinction between reliability and agreement and the need to report both with their uncertainty. Because clinical stability and technical quality could not be independently verified, the observed variation cannot be interpreted as isolated measurement error or used to establish individual clinical-response thresholds.

## Figures and Tables

**Table 1:** Cohort composition and recovered demographic information.

Characteristic	Full Recovered Cohort	Primary Population	Expanded Exploratory Population
Participants, n	72	19	65
Recorded COPD, n (%)	60 (83.3)	14 (73.7)	56 (86.2)
Recorded interstitial lung disease, n (%)	7 (9.7)	2 (10.5)	5 (7.7)
Recorded as healthy, n (%)	5 (6.9)	3 (15.8)	4 (6.2)
Male, n (%)	53 (73.6)	11 (57.9)	48 (73.8)
Female, n (%)	19 (26.4)	8 (42.1)	17 (26.2)
Age and anthropometry available, n/N	26/72	19/19	19/65
Age, years, mean (SD)	60.7 (15.4)	61.7 (16.1)	61.7 (16.1)
Age, years, range	28–83	28–83	28–83
Height, cm, mean (SD)	165.3 (6.5)	164.4 (6.0)	164.4 (6.0)
Weight, kg, mean (SD)	71.5 (15.6)	68.8 (14.4)	68.8 (14.4)
BMI, kg/m^2^, mean (SD)	26.0 (4.8)	25.4 (4.6)	25.4 (4.6)
Dataset-documented separate-day pairs, n	19	19	19
Documented interval, days, median (range)	2 (2–3)	2 (2–3)	2 (2–3)
Same-session pre/post-BD records excluded, n	7	0	0
Complete core pairs retained, n	65	19	65
Paired FRC in retained participants, n	19	19	19

Note. Values are n (%), n/N, mean (SD), median (range), or range, as indicated. Percentages use the column population. Age, height, weight, and BMI are available for 26/72, 19/19, and 19/65 participants, respectively; summaries use these available cases. BMI was calculated from individual weight and height. The primary population is included in the expanded population; their demographic summaries describe the same 19 participants. Diagnostic categories are recorded labels, not independently adjudicated diagnoses; diagnostic post-bronchodilator confirmation of COPD was unavailable. All interval summaries refer to the same 19 dataset-documented separate-day pairs. The seven excluded pre/post-BD records represent single sessions, not zero-day test–retest pairs. According to the principal investigator’s retrospective account, all 65 retained pairs were obtained on different days within six days; exact dates remained unavailable in the dataset for the additional 46. Core pairs comprise TLC, RV, FEV_1_, FVC, and FEV_1_/FVC at both visits in retained participants. Paired FRC was available only in the same 19 primary participants. BD, bronchodilator; BMI, body mass index; COPD, chronic obstructive pulmonary disease; FEV_1_, forced expiratory volume in one second; FRC, functional residual capacity; FVC, forced vital capacity; RV, residual volume; SD, standard deviation; TLC, total lung capacity.

**Table 2:** Paired comparisons, reliability, and concordance for TLC and RV in the primary population.

Statistic	TLC (n = 19)	RV (n = 19)
D1, L, mean (SD)	6.04 (1.44)	3.02 (1.30)
D2, L, mean (SD)	6.01 (1.41)	3.01 (1.35)
Shapiro–Wilk *p* value	0.022	0.311
Paired location test	Wilcoxon signed-rank	Paired *t*-test
Paired location estimate, L (95% CI)	0.010 (−0.180 to 0.170)	−0.015 (−0.241 to 0.211)
Two-sided *p* value	0.763	0.893
ICC(A,1) (95% CI)	0.947 (0.838 to 0.988)	0.940 (0.818 to 0.974)
Lin’s CCC (95% CI)	0.944 (0.831 to 0.988)	0.937 (0.809 to 0.973)

Note. Differences are D2 − D1. Shapiro–Wilk tests were applied to paired differences. The TLC location estimate is the Hodges–Lehmann pseudo median, with an asymptotic signed-rank 95% CI and continuity-corrected *p* value. The RV location estimate is the arithmetic mean difference, with a paired-t 95% CI. These location intervals are distinct from the bootstrap intervals for mean bias in Table 3. ICC(A,1) denotes the two-way mixed-effects, absolute-agreement, single-measure intraclass correlation coefficient. ICC and CCC CIs are percentile intervals from 5000 participant-level bootstrap resamples. *p* values are exploratory and unadjusted for multiplicity. CCC, concordance correlation coefficient; CI, confidence interval; RV, residual volume; SD, standard deviation; TLC, total lung capacity.

**Table 3:** Between-session agreement and descriptive dispersion for TLC and RV in the primary population.

Statistic	TLC (n = 19)	RV (n = 19)
Mean bias, L (95% CI)	−0.033 (−0.256 to 0.161)	−0.015 (−0.219 to 0.190)
Gaussian lower limit, L (95% CI)	−0.964 (−1.511 to −0.364)	−0.934 (−1.293 to −0.501)
Gaussian upper limit, L (95% CI)	0.898 (0.440 to 1.212)	0.904 (0.517 to 1.204)
Empirical 2.5th percentile, L (95% CI)	−1.122 (−1.450 to −0.251)	−0.875 (−1.100 to −0.356)
Empirical 97.5th percentile, L (95% CI)	0.641 (0.251 to 0.870)	0.803 (0.290 to 0.830)
SD_diff_, L (95% CI)	0.475 (0.214 to 0.675)	0.469 (0.288 to 0.603)
Descriptive S_w_, L (95% CI)	0.336 (0.152 to 0.477)	0.332 (0.204 to 0.427)
Descriptive D_95_, L (95% CI)	0.931 (0.420 to 1.323)	0.919 (0.565 to 1.183)
Descriptive CV, % (95% CI)	5.6 (2.5 to 8.1)	11.0 (7.0 to 14.2)
Proportional-bias slope, L/L	−0.021	0.037
Proportional-bias *p* value	0.797	0.678

Note. Bias is the arithmetic mean of D2 − D1. It differs from the TLC Hodges–Lehmann pseudo median in Table 2; under skewness, the estimates can differ in magnitude and sign. All CIs in this table are 95% percentile-bootstrap intervals based on 5000 participant-level resamples. For RV, the point estimate of mean bias equals the paired-t location estimate in Table 2, but the confidence intervals use different methods. Gaussian reference limits equal mean bias ± 1.96 × SD_diff_. Empirical endpoints are type-7 sample quantiles. The CI reported beside each endpoint describes uncertainty in that endpoint, not the range of paired differences. S_w_ = SD_diff_/√2; D_95_ = 1.96 × SD_diff_; CV = 100 × S_w_/grand paired mean. These are descriptive summaries of observed variation, not validated measurement-error estimates or clinical response thresholds. Proportional bias was explored by linear regression of paired differences on pair means; slope *p* values are two-sided and unadjusted. CI, confidence interval; CV, coefficient of variation; RV, residual volume; SD_diff_, standard deviation of paired differences; TLC, total lung capacity.

**Table 4:** Paired comparisons, reliability, and concordance for secondary outcomes in the primary population (n = 19).

Statistic	FEV_1_ (L)	FVC (L)	FEV_1_/FVC (%)	FRC (L)
D1, mean (SD)	1.68 (0.85)	2.91 (0.76)	56.74 (19.96)	4.13 (1.39)
D2, mean (SD)	1.72 (0.89)	2.86 (0.79)	58.32 (19.95)	4.11 (1.53)
Shapiro–Wilk *p* value	0.842	0.044	0.662	0.068
Paired location test	Paired *t*-test	Wilcoxon signed-rank	Paired *t*-test	Paired *t*-test
Paired location estimate (95% CI)	0.036 (−0.037 to 0.109)	−0.048 (−0.200 to 0.120)	1.579 (−0.430 to 3.588)	−0.018 (−0.294 to 0.257)
Two-sided *p* value	0.318	0.513	0.116	0.890
ICC(A,1) (95% CI)	0.985 (0.948 to 0.994)	0.931 (0.816 to 0.970)	0.976 (0.932 to 0.992)	0.927 (0.793 to 0.975)
Lin’s CCC (95% CI)	0.984 (0.945 to 0.994)	0.927 (0.807 to 0.969)	0.975 (0.929 to 0.991)	0.923 (0.784 to 0.973)

Note. All outcomes use the same 19 participants. D1 and D2 are expressed in liters except FEV_1_/FVC, which is expressed as a percentage. Paired location estimates and their CIs are in liters for FEV_1_, FVC, and FRC, and in percentage points for FEV_1_/FVC. ICC and CCC are dimensionless. Differences are D2 − D1. Shapiro–Wilk tests were applied to paired differences. The FVC location estimate is the Hodges–Lehmann pseudo median, with an asymptotic signed-rank 95% CI and continuity-corrected *p* value. Other location estimates are arithmetic mean differences, with paired-t 95% CIs. These intervals are distinct from the bootstrap intervals for mean bias in Table 5. ICC(A,1) denotes the two-way mixed-effects, absolute-agreement, single-measure intraclass correlation coefficient. ICC and CCC CIs are percentile intervals from 5000 participant-level bootstrap resamples. *p* values are exploratory and unadjusted for multiplicity. CCC, concordance correlation coefficient; CI, confidence interval; FEV_1_, forced expiratory volume in one second; FRC, functional residual capacity; FVC, forced vital capacity; SD, standard deviation.

**Table 5:** Between-session agreement and descriptive dispersion for secondary outcomes in the primary population (n = 19).

Statistic	FEV_1_ (L)	FVC (L)	FEV_1_/FVC (pp)	FRC (L)
Mean bias (95% CI)	0.036 (−0.032 to 0.101)	−0.057 (−0.186 to 0.067)	1.579 (−0.263 to 3.368)	−0.018 (−0.284 to 0.223)
Gaussian lower limit (95% CI)	−0.262 (−0.381 to −0.118)	−0.627 (−0.794 to −0.367)	−6.589 (−9.737 to −2.996)	−1.140 (−1.774 to −0.469)
Gaussian upper limit (95% CI)	0.333 (0.224 to 0.419)	0.513 (0.350 to 0.615)	9.747 (5.993 to 12.604)	1.103 (0.574 to 1.467)
Empirical 2.5th percentile (95% CI)	−0.265 (−0.310 to −0.087)	−0.530 (−0.530 to −0.372)	−6.200 (−8.000 to −1.000)	−1.300 (−1.660 to −0.216)
Empirical 97.5th percentile (95% CI)	0.271 (0.136 to 0.320)	0.306 (0.199 to 0.310)	9.100 (5.000 to 10.000)	0.840 (0.340 to 1.020)
SD_diff_ (95% CI)	0.152 (0.096 to 0.193)	0.291 (0.211 to 0.337)	4.168 (2.565 to 5.437)	0.572 (0.285 to 0.800)
Descriptive S_w_ (95% CI)	0.107 (0.068 to 0.136)	0.206 (0.149 to 0.238)	2.947 (1.814 to 3.845)	0.405 (0.202 to 0.566)
Descriptive D_95_ (95% CI)	0.298 (0.188 to 0.378)	0.570 (0.414 to 0.660)	8.168 (5.027 to 10.657)	1.122 (0.559 to 1.568)
Descriptive CV, % (95% CI)	6.3 (3.6 to 9.0)	7.1 (5.0 to 8.5)	5.1 (3.0 to 7.1)	9.8 (5.1 to 14.1)
Proportional-bias slope	0.04224	0.04840	−0.00025	0.09613
Proportional-bias *p* value	0.319	0.605	0.996	0.320

Note. All outcomes use the same 19 participants. Units in column headings apply to bias, limits, SD_diff_, S_w_, and D_95_; CV is a percentage and regression slopes are dimensionless. Bias is the arithmetic mean of D2 − D1; for FVC it differs from the Hodges–Lehmann pseudo median in Table 4. All CIs in this table are 95% percentile-bootstrap intervals from 5000 participant-level resamples. Gaussian reference limits equal mean bias ± 1.96 × SD_diff_. Empirical endpoints are type-7 sample quantiles. Each endpoint CI describes uncertainty in that endpoint, not the range of paired differences. S_w_ = SD_diff_/√2; D_95_ = 1.96 × SD_diff_; CV = 100 × S_w_/grand paired mean. These are descriptive summaries of observed variation, not validated measurement-error estimates or clinical response thresholds. Proportional bias was explored by linear regression of paired differences on pair means; slope *p* values are two-sided and unadjusted. CI, confidence interval; CV, coefficient of variation; FEV_1_, forced expiratory volume in one second; FRC, functional residual capacity; FVC, forced vital capacity; pp, percentage points; SD_diff_, standard deviation of paired differences.

## Data Availability

Participant-level data are not publicly available because the consent obtained concerns scientific reuse of clinical information and dissemination of aggregate findings. Aggregate statistical results, variable definitions, and analysis code accompany the manuscript. Requests concerning access to participant-level data may be directed to the corresponding author and will be assessed against the applicable consent and data-protection requirements.

## Supplementary Materials

The following supporting information can be downloaded at: https://www.mdpi.com/article/10.3390/jcm15197704/s1, Table S1. Expanded exploratory analysis; Table S2. Analyses restricted to participants with a recorded COPD label; Table S3. Baseline spirometric and lung-volume measurements in the analysis populations and recorded-COPD subgroups; Table S4. GRRAS item-by-item reporting audit; Figure S1. Aggregate agreement and influence summaries for functional residual capacity (FRC) in the primary population (n = 19).

## Abbreviations

ATS, American Thoracic Society; BD, bronchodilator; BMI, body mass index; CCC, concordance correlation coefficient; CI, confidence interval; COPD, chronic obstructive pulmonary disease; CV, coefficient of variation; D1, first examination; D2, second examination; D_95_, descriptive half-width of the Gaussian reference limits (1.96 × SDdiff); ERS, European Respiratory Society; EU, European Union; FEV_1_, forced expiratory volume in one second; FEV_1_/FVC, ratio of forced expiratory volume in one second to forced vital capacity; FRC, functional residual capacity; FVC, forced vital capacity; GDPR, General Data Protection Regulation; GLI, Global Lung Function Initiative; GOLD, Global Initiative for Chronic Obstructive Lung Disease; GRRAS, Guidelines for Reporting Reliability and Agreement Studies; HL, Hodges–Lehmann pseudo median; ICC, intraclass correlation coefficient; ICC(A,1), absolute-agreement, single-measure intraclass correlation coefficient; ILD, interstitial lung disease; LoA, limits of agreement; MDC_95_, minimal detectable change at the 95% confidence level; pp, percentage points; RV, residual volume; RV/TLC, ratio of residual volume to total lung capacity; SD, standard deviation; SDdiff, standard deviation of paired differences; Sw, descriptive within-participant dispersion (SDdiff/√2); TLC, total lung capacity.
