# Supplementary material for: Between-Session Reliability and Agreement of Body-Plethysmographic Lung Volumes in a Legacy Pulmonary Function Cohort
Source: J Clin Med. 2026 Oct 5;15(19):7704. doi: 10.3390/jcm15197704 (PMC13666337; doi:10.3390/jcm15197704)
Supplement: Supplementary file 1 [file jcm-15-07704-s001.zip › jcm-4569685-supplementary.pdf]

Supplementary Material

Table S1. Expanded exploratory analysis. Panel A: static lung volumes.

Statistic	TLC (L)	RV (L)	FRC (L)
Paired observations, n	65	65	19
D1, mean (SD)	5.59 (1.41)	3.04 (1.20)	4.13 (1.39)
D2, mean (SD)	5.53 (1.29)	2.95 (1.11)	4.11 (1.53)
Paired location estimate (95% CI)	HL: 0.005 (−0.090 to 0.090)	HL: −0.030 (−0.120 to 0.055)	Mean: −0.018 (−0.294 to 0.257)
Paired location p value	0.901	0.482	0.890
ICC(A,1) (95% CI)	0.917 (0.847 to 0.965)	0.883 (0.782 to 0.946)	0.927 (0.793 to 0.975)
Lin's CCC (95% CI)	0.916 (0.845 to 0.964)	0.882 (0.779 to 0.945)	0.923 (0.784 to 0.973)
Mean bias (95% CI)	−0.062 (−0.205 to 0.061)	−0.089 (−0.226 to 0.033)	−0.018 (−0.284 to 0.223)
Gaussian reference limits	−1.143 to 1.019	−1.180 to 1.002	−1.140 to 1.103
Empirical 2.5th–97.5th percentiles	−1.546 to 0.834	−1.276 to 0.790	−1.300 to 0.840
SD_{diff} (95% CI)	0.551 (0.351 to 0.740)	0.557 (0.375 to 0.733)	0.572 (0.285 to 0.800)
Descriptive S_w (95% CI)	0.390 (0.249 to 0.523)	0.394 (0.265 to 0.519)	0.405 (0.202 to 0.566)
Descriptive D_{95} (95% CI)	1.081 (0.689 to 1.450)	1.091 (0.735 to 1.438)	1.122 (0.559 to 1.568)
Descriptive CV, % (95% CI)	7.0 (4.5 to 9.3)	13.2 (9.1 to 16.9)	9.8 (5.1 to 14.1)
Proportional-bias slope	−0.09566	−0.07986	0.09613
Proportional-bias p value	0.065	0.200	0.320

Note. The expanded population (n = 65) includes all 19 primary participants; comparisons with primary results in Tables 2–5 are descriptive, not independent-group comparisons. FRC uses only those same 19 participants and is not independent replication. Units are liters, except dimensionless coefficients and CV (%). Differences are D2 − D1. "Mean" denotes a paired-t estimate and CI; "HL" denotes the Hodges–Lehmann pseudo median with an asymptotic signed-rank CI and continuity-corrected p value. Mean bias is always the arithmetic mean difference. ICC(A,1) uses a two-way mixed-effects, absolute-agreement, single-measure model. All other CIs shown are 95% percentile-bootstrap intervals from 5000 paired participant-level resamples. Gaussian limits = mean bias ± 1.96 × SD_{diff}; empirical endpoints are type-7 quantiles. These ranges are not CIs. $S_w = SD_{diff}/\sqrt{2}$; $D_{95} = 1.96 \times SD_{diff}$; CV = 100 × S_w/grand paired mean. Dispersion is descriptive, not a validated clinical threshold. Slope tests regress differences on pair means; slopes are dimensionless. Paired location and slope tests are two-sided and unadjusted. Endpoint CIs and Shapiro–Wilk results are in Supplementary Data S1. CCC, concordance correlation coefficient; CI, confidence interval; CV, coefficient of variation; FRC, functional residual capacity; ICC, intraclass correlation coefficient; RV, residual volume; SD_{diff}, SD of paired differences; TLC, total lung capacity.

Table S1. (continued) Expanded exploratory analysis. Panel B: spirometric outcomes.

Statistic	FEV_1 (L)	FVC (L)	FEV_1/FVC (%, pp)
Paired observations, n	65	65	65
D1, mean (SD)	1.32 (0.68)	2.44 (0.74)	52.78 (15.30)
D2, mean (SD)	1.34 (0.69)	2.46 (0.72)	53.37 (16.25)
Paired location estimate (95% CI)	Mean: 0.028 (−0.001 to 0.056)	HL: 0.035 (−0.020 to 0.080)	Mean: 0.585 (−0.334 to 1.503)
Paired location p value	0.056	0.205	0.208
ICC(A,1) (95% CI)	0.985 (0.966 to 0.993)	0.948 (0.908 to 0.970)	0.972 (0.949 to 0.984)
Lin's CCC (95% CI)	0.985 (0.965 to 0.992)	0.947 (0.907 to 0.969)	0.972 (0.948 to 0.984)
Mean bias (95% CI)	0.028 (−0.001 to 0.056)	0.016 (−0.041 to 0.074)	0.585 (−0.308 to 1.462)
Gaussian reference limits	−0.197 to 0.253	−0.446 to 0.479	−6.682 to 7.852
Empirical 2.5th–97.5th percentiles	−0.230 to 0.254	−0.530 to 0.404	−6.800 to 8.800
SD_{diff} (95% CI)	0.115 (0.088 to 0.139)	0.236 (0.188 to 0.276)	3.708 (2.950 to 4.401)
Descriptive S_w (95% CI)	0.081 (0.062 to 0.099)	0.167 (0.133 to 0.195)	2.622 (2.086 to 3.112)
Descriptive D_{95} (95% CI)	0.225 (0.173 to 0.273)	0.463 (0.369 to 0.541)	7.267 (5.782 to 8.627)
Descriptive CV, % (95% CI)	6.1 (4.6 to 7.6)	6.8 (5.4 to 8.1)	4.9 (3.8 to 6.0)

Statistic	FEV_1 (L)	FVC (L)	FEV_1/FVC (%, pp)
Proportional-bias slope	0.01529	−0.03289	0.06093
Proportional-bias p value	0.471	0.428	0.038

Note. The expanded population (n = 65) includes all 19 primary participants; comparisons with primary results in Tables 2–5 are descriptive, not independent-group comparisons. FEV_1/FVC levels at D1 and D2 are percentages; changes and dispersion are percentage points. Volumes are in liters; ICC and CCC are dimensionless. Differences are D2 − D1. "Mean" denotes a paired-t estimate and CI; "HL" denotes the Hodges–Lehmann pseudomedian with an asymptotic signed-rank CI and continuity-corrected p value. Mean bias is always the arithmetic mean difference. ICC(A,1) uses a two-way mixed-effects, absolute-agreement, single-measure model. All other CIs shown are 95% percentile-bootstrap intervals from 5000 paired participant-level resamples. Gaussian limits = mean bias ± 1.96 × SD_{diff}; empirical endpoints are type-7 quantiles. These ranges are not CIs. S_w = $SD_{diff}/\sqrt{2}$; D_{95} = 1.96 × SD_{diff}; CV = 100 × S_w/grand paired mean. Dispersion is descriptive, not a validated clinical threshold. Slope tests regress differences on pair means; slopes are dimensionless. Paired location and slope tests are two-sided and unadjusted. Endpoint CIs and Shapiro–Wilk results are in Supplementary Data S1. CCC, concordance correlation coefficient; CI, confidence interval; CV, coefficient of variation; FEV_1, forced expiratory volume in one second; FVC, forced vital capacity; ICC, intraclass correlation coefficient; pp, percentage points; SD_{diff}, SD of paired differences.

Table S2. Analyses restricted to participants with a recorded COPD label. Panel A: static lung volumes in the primary population (n = 14).

Statistic	TLC (L)	RV (L)	FRC (L)
Paired observations, n	14	14	14
D1, mean (SD)	6.51 (1.37)	3.50 (1.17)	4.58 (1.28)
D2, mean (SD)	6.39 (1.43)	3.47 (1.26)	4.65 (1.41)
Paired location estimate (95% CI)	HL: −0.010 (−0.330 to 0.175)	Mean: −0.029 (−0.306 to 0.249)	Mean: 0.071 (−0.189 to 0.332)
Paired location p value	0.900	0.827	0.564
ICC(A,1) (95% CI)	0.940 (0.788 to 0.990)	0.927 (0.652 to 0.972)	0.946 (0.855 to 0.976)
Lin's CCC (95% CI)	0.936 (0.775 to 0.989)	0.922 (0.635 to 0.969)	0.942 (0.845 to 0.975)
Mean bias (95% CI)	−0.112 (−0.378 to 0.101)	−0.029 (−0.282 to 0.200)	0.071 (−0.160 to 0.301)
Gaussian reference limits	−1.067 to 0.843	−0.971 to 0.914	−0.814 to 0.957
Empirical 2.5th–97.5th percentiles	−1.213 to 0.347	−0.938 to 0.749	−0.724 to 0.877
SD_{diff} (95% CI)	0.487 (0.204 to 0.687)	0.481 (0.245 to 0.640)	0.452 (0.230 to 0.605)
Descriptive S_w (95% CI)	0.345 (0.145 to 0.486)	0.340 (0.173 to 0.452)	0.319 (0.162 to 0.428)

Statistic	TLC (L)	RV (L)	FRC (L)
Descriptive D_{95} (95% CI)	0.955 (0.401 to 1.347)	0.942 (0.480 to 1.254)	0.885 (0.450 to 1.187)
Descriptive CV, % (95% CI)	5.3 (2.3 to 7.8)	9.8 (5.3 to 12.7)	6.9 (3.7 to 8.9)
Proportional-bias slope	0.04390	0.07777	0.09453
Proportional-bias p value	0.672	0.509	0.336

Note. COPD is a recorded label without independently verified diagnostic post-bronchodilator confirmation. The 14 primary participants are included in the 56 expanded participants; these are overlapping, exploratory subgroups. FRC is based on the same 14 participants in both subgroups. Units are liters, except dimensionless coefficients and CV (%). Differences are D2 − D1. "Mean" denotes a paired-t estimate and CI; "HL" denotes the Hodges–Lehmann pseudomedian with an asymptotic signed-rank CI and continuity-corrected p value. Mean bias is always the arithmetic mean difference. ICC(A,1) uses a two-way mixed-effects, absolute-agreement, single-measure model. All other CIs shown are 95% percentile-bootstrap intervals from 5000 paired participant-level resamples. Gaussian limits = mean bias ± 1.96 × SD_{diff}; empirical endpoints are type-7 quantiles. These ranges are not CIs. S_w = $SD_{diff}/\sqrt{2}$; D_{95} = 1.96 × SD_{diff}; CV = 100 × S_w/grand paired mean. Dispersion is descriptive, not a validated clinical threshold. Slope tests regress differences on pair means; slopes are dimensionless. Paired location and slope tests are two-sided and unadjusted. Endpoint CIs and Shapiro–Wilk results are in Supplementary Data S1. COPD, chronic obstructive pulmonary disease; CCC, concordance correlation coefficient; CI, confidence interval; CV, coefficient of variation; FRC, functional residual capacity; ICC, intraclass correlation coefficient; RV, residual volume; SD_{diff}, SD of paired differences; TLC, total lung capacity.

Table S2. (continued) Analyses restricted to participants with a recorded COPD label. Panel B: spirometric outcomes in the primary population (n = 14).

Statistic	FEV_1 (L)	FVC (L)	FEV_1/FVC (%, pp)
Paired observations, n	14	14	14
D1, mean (SD)	1.48 (0.78)	2.96 (0.78)	48.86 (14.98)
D2, mean (SD)	1.52 (0.84)	2.85 (0.85)	51.36 (15.61)
Paired location estimate (95% CI)	Mean: 0.041 (−0.056 to 0.138)	Mean: −0.105 (−0.289 to 0.079)	Mean: 2.500 (0.227 to 4.773)
Paired location p value	0.381	0.239	0.034
ICC(A,1) (95% CI)	0.979 (0.842 to 0.993)	0.922 (0.759 to 0.969)	0.956 (0.852 to 0.986)
Lin's CCC (95% CI)	0.977 (0.832 to 0.993)	0.916 (0.745 to 0.967)	0.953 (0.842 to 0.985)
Mean bias (95% CI)	0.041 (−0.049 to 0.121)	−0.105 (−0.267 to 0.052)	2.500 (0.571 to 4.500)
Gaussian reference limits	−0.289 to 0.370	−0.729 to 0.519	−5.217 to 10.217

Statistic	FEV_1 (L)	FVC (L)	FEV_1/FVC (%, pp)
Empirical 2.5th–97.5th percentiles	−0.278 to 0.284	−0.530 to 0.307	−3.025 to 9.350
SD_{diff} (95% CI)	0.168 (0.094 to 0.217)	0.319 (0.234 to 0.363)	3.937 (2.499 to 4.881)
Descriptive S_w (95% CI)	0.119 (0.066 to 0.153)	0.225 (0.165 to 0.257)	2.784 (1.767 to 3.451)
Descriptive D_{95} (95% CI)	0.329 (0.183 to 0.425)	0.624 (0.458 to 0.712)	7.717 (4.899 to 9.567)
Descriptive CV, % (95% CI)	7.9 (4.0 to 11.4)	7.8 (5.5 to 9.1)	5.6 (3.5 to 7.1)
Proportional-bias slope	0.07929	0.08841	0.04201
Proportional-bias p value	0.179	0.443	0.580

Note. COPD is a recorded label without independently verified diagnostic post-bronchodilator confirmation. The 14 primary participants are included in the 56 expanded participants; these are overlapping, exploratory subgroups. FEV_1/FVC levels are percentages; changes and dispersion are percentage points. Volumes are in liters; ICC and CCC are dimensionless. Differences are D2 − D1. "Mean" denotes a paired-t estimate and CI; "HL" denotes the Hodges–Lehmann pseudomedian with an asymptotic signed-rank CI and continuity-corrected p value. Mean bias is always the arithmetic mean difference. ICC(A,1) uses a two-way mixed-effects, absolute-agreement, single-measure model. All other CIs shown are 95% percentile-bootstrap intervals from 5000 paired participant-level resamples. Gaussian limits = mean bias ± 1.96 × SD_{diff}; empirical endpoints are type-7 quantiles. These ranges are not CIs. $S_w = SD_{diff}/\sqrt{2}$; $D_{95} = 1.96 \times SD_{diff}$; CV = 100 × S_w/grand paired mean. Dispersion is descriptive, not a validated clinical threshold. Slope tests regress differences on pair means; slopes are dimensionless. Paired location and slope tests are two-sided and unadjusted. Endpoint CIs and Shapiro–Wilk results are in Supplementary Data S1. COPD, chronic obstructive pulmonary disease; CCC, concordance correlation coefficient; CI, confidence interval; CV, coefficient of variation; FEV_1, forced expiratory volume in one second; FVC, forced vital capacity; ICC, intraclass correlation coefficient; pp, percentage points; SD_{diff}, SD of paired differences.

Table S2. (continued) Analyses restricted to participants with a recorded COPD label. Panel C: static lung volumes in the expanded exploratory population (n = 56).

Statistic	TLC (L)	RV (L)	FRC (L)
Paired observations, n	56	56	14
D1, mean (SD)	5.76 (1.42)	3.25 (1.13)	4.58 (1.28)
D2, mean (SD)	5.68 (1.29)	3.15 (1.04)	4.65 (1.41)
Paired location estimate (95% CI)	HL: 0.005 (−0.120 to 0.095)	HL: −0.040 (−0.150 to 0.070)	Mean: 0.071 (−0.189 to 0.332)
Paired location p value	0.961	0.514	0.564
ICC(A,1) (95% CI)	0.910 (0.828 to 0.964)	0.853 (0.717 to 0.936)	0.946 (0.855 to 0.976)

Statistic	TLC (L)	RV (L)	FRC (L)
Lin's CCC (95% CI)	0.908<br>(0.825 to 0.963)	0.851<br>(0.713 to 0.934)	0.942<br>(0.845 to 0.975)
Mean bias (95% CI)	−0.081<br>(−0.240 to 0.056)	−0.104<br>(−0.264 to 0.039)	0.071<br>(−0.160 to 0.301)
Gaussian reference limits	−1.211 to 1.050	−1.250 to 1.041	−0.814 to 0.957
Empirical 2.5th–97.5th percentiles	−1.600 to 0.763	−1.353 to 0.745	−0.724 to 0.877
SD_{diff} (95% CI)	0.577<br>(0.357 to 0.780)	0.584<br>(0.393 to 0.780)	0.452<br>(0.230 to 0.605)
Descriptive S_w (95% CI)	0.408<br>(0.252 to 0.551)	0.413<br>(0.278 to 0.552)	0.319<br>(0.162 to 0.428)
Descriptive D_{95} (95% CI)	1.131<br>(0.700 to 1.528)	1.146<br>(0.770 to 1.529)	0.885<br>(0.450 to 1.187)
Descriptive CV, % (95% CI)	7.1<br>(4.5 to 9.5)	12.9<br>(8.8 to 16.9)	6.9<br>(3.7 to 8.9)
Proportional-bias slope	−0.10267	−0.08396	0.09453
Proportional-bias p value	0.079	0.270	0.336

Note. COPD is a recorded label without independently verified diagnostic post-bronchodilator confirmation. The 14 primary participants are included in the 56 expanded participants; these are overlapping, exploratory subgroups. FRC is based on the same 14 participants in both subgroups. Units are liters, except dimensionless coefficients and CV (%). Differences are D2 − D1. "Mean" denotes a paired-t estimate and CI; "HL" denotes the Hodges–Lehmann pseudomedian with an asymptotic signed-rank CI and continuity-corrected p value. Mean bias is always the arithmetic mean difference. ICC(A,1) uses a two-way mixed-effects, absolute-agreement, single-measure model. All other CIs shown are 95% percentile-bootstrap intervals from 5000 paired participant-level resamples. Gaussian limits = mean bias ± 1.96 × SD_{diff}; empirical endpoints are type-7 quantiles. These ranges are not CIs. $S_w = SD_{diff}/\sqrt{2}$; $D_{95} = 1.96 \times SD_{diff}$; CV = 100 × S_w/grand paired mean. Dispersion is descriptive, not a validated clinical threshold. Slope tests regress differences on pair means; slopes are dimensionless. Paired location and slope tests are two-sided and unadjusted. Endpoint CIs and Shapiro–Wilk results are in Supplementary Data S1. COPD, chronic obstructive pulmonary disease; CCC, concordance correlation coefficient; CI, confidence interval; CV, coefficient of variation; FRC, functional residual capacity; ICC, intraclass correlation coefficient; RV, residual volume; SD_{diff}, SD of paired differences; TLC, total lung capacity.

Table S2. (continued) Analyses restricted to participants with a recorded COPD label. Panel D: spirometric outcomes in the expanded exploratory population (n = 56).

Statistic	FEV_1 (L)	FVC (L)	FEV_1/FVC (%, pp)
Paired observations, n	56	56	56
D1, mean (SD)	1.17 (0.52)	2.39 (0.70)	48.70 (11.08)
D2, mean (SD)	1.21 (0.54)	2.40 (0.68)	49.38 (12.49)

Statistic	FEV_1 (L)	FVC (L)	FEV_1/FVC (%, pp)
Paired location estimate (95% CI)	Mean: 0.031 (−0.001 to 0.063)	HL: 0.035 (−0.040 to 0.090)	Mean: 0.679 (−0.339 to 1.696)
Paired location p value	0.059	0.259	0.187
ICC(A,1) (95% CI)	0.973 (0.924 to 0.989)	0.935 (0.881 to 0.963)	0.948 (0.909 to 0.971)
Lin's CCC (95% CI)	0.973 (0.923 to 0.988)	0.934 (0.879 to 0.963)	0.947 (0.908 to 0.970)
Mean bias (95% CI)	0.031 (0.001 to 0.063)	0.015 (−0.051 to 0.078)	0.679 (−0.268 to 1.661)
Gaussian reference limits	−0.204 to 0.266	−0.476 to 0.506	−6.769 to 8.127
Empirical 2.5th–97.5th percentiles	−0.241 to 0.279	−0.530 to 0.418	−6.000 to 9.250
SD_{diff} (95% CI)	0.120 (0.090 to 0.146)	0.251 (0.199 to 0.293)	3.800 (2.994 to 4.519)
Descriptive S_w (95% CI)	0.085 (0.064 to 0.103)	0.177 (0.140 to 0.207)	2.687 (2.117 to 3.195)
Descriptive D_{95} (95% CI)	0.235 (0.177 to 0.287)	0.491 (0.389 to 0.573)	7.448 (5.868 to 8.856)
Descriptive CV, % (95% CI)	7.1 (5.3 to 8.8)	7.4 (5.8 to 8.7)	5.5 (4.3 to 6.5)
Proportional-bias slope	0.04839	−0.03798	0.12271
Proportional-bias p value	0.115	0.451	0.004

Note. COPD is a recorded label without independently verified diagnostic post-bronchodilator confirmation. The 14 primary participants are included in the 56 expanded participants; these are overlapping, exploratory subgroups. FEV_1/FVC levels are percentages; changes and dispersion are percentage points. Volumes are in liters; ICC and CCC are dimensionless. Differences are D2 − D1. "Mean" denotes a paired-t estimate and CI; "HL" denotes the Hodges–Lehmann pseudomedian with an asymptotic signed-rank CI and continuity-corrected p value. Mean bias is always the arithmetic mean difference. ICC(A,1) uses a two-way mixed-effects, absolute-agreement, single-measure model. All other CIs shown are 95% percentile-bootstrap intervals from 5000 paired participant-level resamples. Gaussian limits = mean bias ± 1.96 × SD_{diff}; empirical endpoints are type-7 quantiles. These ranges are not CIs. $S_w = SD_{diff}/\sqrt{2}$; $D_{95} = 1.96 \times SD_{diff}$; CV = 100 × S_w/grand paired mean. Dispersion is descriptive, not a validated clinical threshold. Slope tests regress differences on pair means; slopes are dimensionless. Paired location and slope tests are two-sided and unadjusted. Endpoint CIs and Shapiro–Wilk results are in Supplementary Data S1. COPD, chronic obstructive pulmonary disease; CCC, concordance correlation coefficient; CI, confidence interval; CV, coefficient of variation; FEV_1, forced expiratory volume in one second; FVC, forced vital capacity; ICC, intraclass correlation coefficient; pp, percentage points; SD_{diff}, SD of paired differences.

Table S3. Baseline spirometric and lung-volume measurements in the analysis populations and recorded-COPD subgroups.

D1 measurement	Primary (n = 19)	Expanded (n = 65)	Recorded COPD primary (n = 14)	Recorded COPD expanded (n = 56)
FEV_1, L	1.68 (0.85)<br>1.49<br>(0.64 to 3.69)	1.32 (0.68)<br>1.08<br>(0.50 to 3.69)	1.48 (0.78)<br>1.32<br>(0.64 to 3.69)	1.17 (0.52)<br>1.02<br>(0.50 to 3.69)
FVC, L	2.91 (0.76)<br>2.75<br>(1.72 to 4.52)	2.44 (0.74)<br>2.40<br>(1.25 to 4.52)	2.96 (0.78)<br>2.77<br>(1.72 to 4.52)	2.39 (0.70)<br>2.40<br>(1.25 to 4.52)
FEV_1/FVC, %	56.7 (20.0)<br>54.0<br>(25.0 to 95.0)	52.8 (15.3)<br>51.0<br>(25.0 to 95.0)	48.9 (15.0)<br>50.5<br>(25.0 to 82.0)	48.7 (11.1)<br>48.5<br>(25.0 to 82.0)
RV, L	3.02 (1.30)<br>2.83<br>(1.20 to 6.19)	3.04 (1.20)<br>2.73<br>(0.51 to 6.41)	3.50 (1.17)<br>3.47<br>(2.19 to 6.19)	3.25 (1.13)<br>2.85<br>(1.04 to 6.41)
TLC, L	6.04 (1.44)<br>6.40<br>(4.08 to 8.71)	5.59 (1.41)<br>5.34<br>(3.03 to 9.14)	6.51 (1.37)<br>6.71<br>(4.18 to 8.71)	5.76 (1.42)<br>5.51<br>(3.72 to 9.14)
RV/TLC, %	48.7 (11.8)<br>50.3<br>(23.4 to 71.1)	53.4 (12.0)<br>54.4<br>(10.8 to 72.2)	53.2 (9.4)<br>51.5<br>(34.5 to 71.1)	56.0 (9.7)<br>56.4<br>(24.0 to 72.2)

Note. Each cell reports mean (SD), followed by median and (minimum to maximum). All measurements refer to D1; there are no missing values for the six variables within the populations shown. Column denominators apply to every row. The primary population is contained within the expanded population, and the recorded-COPD groups are subsets of these respective populations. No independent-group tests were performed. FEV_1/FVC is the recorded ratio on a 0–100 scale and was not reconstructed from rounded volumes. RV/TLC was calculated for each participant as 100 × RV/TLC, and those individual ratios were summarized; it is not the ratio of group means. These descriptive values do not establish GOLD grades, confirmed post-bronchodilator obstruction, or reference-based hyperinflation categories. No percent-predicted values or z-scores are presented. COPD, chronic obstructive pulmonary disease; FEV_1, forced expiratory volume in one second; FVC, forced vital capacity; RV, residual volume; SD, standard deviation; TLC, total lung capacity.

Table S4. GRRAS item-by-item reporting audit. Panel A: items 1–5.

Item	Reporting topic	Available information, evidence level, and manuscript location
1	Identify reliability or agreement in the title or abstract.	Partially reportable. The title and Abstract identify between-session reliability and agreement in a legacy cohort. Operator identity is unavailable, so the intra- or inter-operator classification requested by GRRAS cannot be established. Location: Title; Abstract; Sections 2.2–2.3 and 5.
2	Describe the measurement instrument.	Partially reportable. The dataset thesis names the MasterScope Body system (JAEGER, Hoechberg, Germany) and "Spirometry Filter 74". This is dataset-thesis information, not session-level verification of equipment, calibration, or software. Software version and technical logs are unavailable. Location: Introduction; Section 2.2; Section 5.
3	Define the participant population of interest.	Reported with diagnostic limitations. The recovered clinical dataset contains 72 participants; 65 remain after excluding seven explicit same-session pre/post-bronchodilator records. The 19 dataset-documented separate-day pairs form the primary population. Diagnostic categories are recorded labels, not independently adjudicated diagnoses. Location: Introduction; Sections 2.1 and 3.1; Figure 1; Table 1.
4	Define the relevant operator population.	Not reportable from the available records. Session-level operator identity, number, qualifications, training, and experience are unavailable. No inference across operators or laboratories is made. The investigator's retrospective account of examination conditions does not supply operator-level records. Location: Sections 2.2–2.3 and 5.
5	Explain prior evidence and the study rationale.	Reported. The Introduction distinguishes reliability from agreement, summarizes the rationale for examining repeated lung-volume measurements, and defines the exploratory purpose. The Discussion compares observed variation with relevant literature without treating other studies as validation of this dataset. Location: Introduction; Section 4.

Note. Topics are concise paraphrases of the 15 GRRAS items. "Reported" refers to reporting, not certification of design, technical quality, or clinical stability. "Partially reportable" and "unverifiable" identify substantive gaps. Dataset records, the dataset thesis, retrospective investigator clarification, patient accounts, and unavailable information are distinguished above.

GRRAS dataset: Kottner J, Audigé L, Brorson S, et al. Guidelines for Reporting Reliability and Agreement Studies (GRRAS) were proposed. J Clin Epidemiol. 2011;64:96–106. doi:10.1016/j.jclinepi.2010.03.002 (reference 8 in the manuscript).

Table S4. (continued) GRRAS item-by-item reporting audit. Panel B: items 6–10.

Item	Reporting topic	Available information, evidence level, and manuscript location
6	Explain sample size and planned observation numbers.	Partially reportable. Sample size was determined by recoverable historical records; no prospective sample-size calculation or dated prespecified analysis plan was available. Each retained participant contributes two session-level values per available outcome. The operator count is unavailable. Location: Sections 2.1 and 2.4; Sections 3.1 and 5; Figure 1; Table 1.
7	Describe how participants and operators were sampled.	Partially reportable. This is a secondary analysis of a fixed, available legacy dataset, with inclusion determined by record availability and measurement context. Consecutive or random recruitment is not established; operator sampling cannot be reconstructed. Dataset-document selection may affect representativeness. Location: Sections 2.1 and 5; Figure 1.
8	Describe measurement conditions, timing, information, and blinding.	Partially reportable. Dataset records document paired pre-bronchodilator labels and intervals of two days in 18 primary pairs and three days in one. Separate days within six days for all 65, comparable bronchodilator conditions for the additional 46, and absence of clinical changes rely on retrospective investigator clarification; unchanged usual therapy relies on patient accounts. Exact dates for 46, technical-quality records, information access, and blinding are unverifiable. Location: Sections 2.1–2.2, 3.1, and 5.
9	Establish whether measurements were independent.	Unverifiable. The records do not establish whether operators had access to prior results or whether measurement decisions were independent. Examinations occurring on separate days do not establish independence or blinding. Location: Sections 2.2 and 5.
10	Specify the statistical methods.	Reported. Outcomes, paired differences, ICC(A,1), CCC, mean bias, Gaussian and empirical limits, descriptive dispersion, paired location tests, proportional-bias regressions, paired bootstrap uncertainty, missing-data handling, overlapping populations, and leave-one-out analyses are specified. Software version and random seed are reported. Location: Sections 2.3–2.4; table notes; Supplementary Data S1.

Note. Topics are concise paraphrases of the 15 GRRAS items. “Reported” refers to reporting, not certification of design, technical quality, or clinical stability. “Partially reportable” and “unverifiable” identify substantive gaps. Dataset records, the dataset thesis, retrospective investigator clarification, patient accounts, and unavailable information are distinguished above.

Checklist dataset: Kottner et al. (2011), manuscript reference 8; see panel A for the full citation.

Table S4. (continued) GRRAS item-by-item reporting audit. Panel C: items 11–15.

Item	Reporting topic	Available information, evidence level, and manuscript location
11	Report achieved participant, operator, and repeat counts.	Partially reportable. There are 19 primary and 65 expanded participants, with two values each for the five core outcomes. FRC has two values for only the same 19 primary participants. Recorded-COPD subsets contain 14 and 56 participants, respectively; paired FRC exists only for the same 14. Operator and within-session maneuver counts are unavailable. Location: Sections 2.1–2.2 and 3.1; Figure 1; Tables 1 and S1–S2.
12	Describe participant and operator characteristics.	Partially reportable. Recorded diagnoses and sex are available for retained participants. Age, height, weight, and BMI are available for all 19 primary participants but not for the additional 46. Baseline physiological summaries are reported; smoking, detailed medication regimens, comorbidities, verified disease severity, and operator characteristics are unavailable. Location: Sections 2.2, 3.1, and 5; Tables 1 and S3.
13	Present estimates with statistical uncertainty.	Reported with limitations of precision. ICC and CCC, paired location estimates, mean bias, Gaussian and empirical limits, and descriptive dispersion are presented with the specified uncertainty methods. Endpoint CIs are in main agreement tables and Supplementary Data S1. Small samples, exploratory unadjusted testing, and leave-one-out sensitivity constrain interpretation. Location: Sections 3.2–3.5; Tables 2–5 and S1–S2; Figures 2–3 and S1; Supplementary Data S1.
14	Interpret practical implications and limitations.	Reported. The Discussion and limitations distinguish relative reliability from agreement in physical units. Technical, biological, and potentially clinical contributions cannot be separated. D_{95} is descriptive, not a validated minimal detectable change or clinical response threshold; nonsignificant paired tests do not show equivalence. Location: Sections 4–6.
15	Make detailed supporting results accessible.	Reported with an access limitation. Supplementary tables and Figure S1 provide aggregate results. Supplementary Data S1 contains machine-readable aggregate outputs, variable definitions, analysis code, and instructions. Participant-level inputs remain restricted, so public aggregate material alone does not permit independent rerunning of every participant-level analysis. Access requests are subject to applicable consent and data-protection conditions. Location: Supplementary Material; Data Availability Statement.

Note. Topics are concise paraphrases of the 15 GRRAS items. “Reported” refers to reporting, not certification of design, technical quality, or clinical stability. “Partially reportable” and “unverifiable” identify substantive gaps. Dataset records, the dataset thesis, retrospective investigator clarification, patient accounts, and unavailable information are distinguished above.

Checklist dataset: Kottner et al. (2011), manuscript reference 8; see panel A for the full citation.

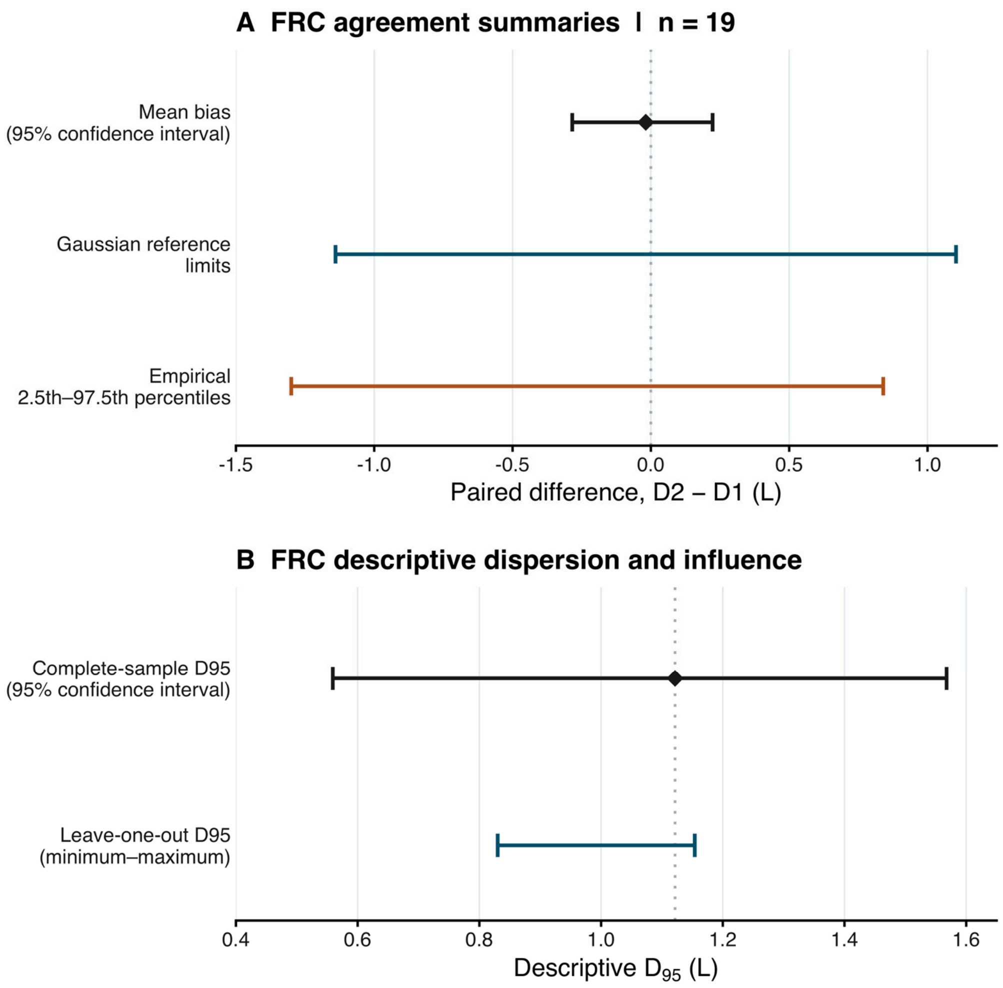

Figure S1. Aggregate agreement and influence summaries for functional residual capacity (FRC) in the primary population (n = 19). (A) The black diamond shows mean bias (D2 − D1), with its 95% percentile-bootstrap CI. Blue and orange bars span the Gaussian reference limits and empirical 2.5th–97.5th percentiles, respectively; these two bars describe ranges of differences, not CIs. (B) The black diamond shows complete-sample descriptive D_{95}, 1.122 L, with its 95% bootstrap CI (0.559–1.568 L). The blue bar spans the leave-one-out D_{95} estimates (0.830–1.154 L); it is a sensitivity range, not a CI. The largest reduction from the complete-sample estimate was 26.0%. The dotted vertical line in panel B marks the complete-sample D_{95}. Each leave-one-out fit uses 18 pairs; all 19 are retained in the principal estimates. Bootstrap intervals use 5000 participant-level resamples. FRC observations are identical in the primary and expanded analyses. No individual records are displayed. D_{95} = 1.96 × SD_{diff} is descriptive, not a validated clinical response threshold; neither range establishes 95% coverage for future differences. CI, confidence interval; SD_{diff}, standard deviation of paired differences.
